# High-LET charged particles: radiobiology and application for new approaches in radiotherapy

**DOI:** 10.1007/s00066-023-02158-7

**Published:** 2023-10-23

**Authors:** Alexander Helm, Claudia Fournier

**Affiliations:** Biophysics Department, GSI Helmholtz Center for Heavy Ion Research, Darmstadt, Germany

**Keywords:** Bragg peak, Relative biological effectiveness, Carbon ions, Combined therapies, Immunogenicity

## Abstract

The number of patients treated with charged-particle radiotherapy as well as the number of treatment centers is increasing worldwide, particularly regarding protons. However, high-linear energy transfer (LET) particles, mainly carbon ions, are of special interest for application in radiotherapy, as their special physical features result in high precision and hence lower toxicity, and at the same time in increased efficiency in cell inactivation in the target region, i.e., the tumor. The radiobiology of high-LET particles differs with respect to DNA damage repair, cytogenetic damage, and cell death type, and their increased LET can tackle cells’ resistance to hypoxia. Recent developments and perspectives, e.g., the return of high-LET particle therapy to the US with a center planned at Mayo clinics, the application of carbon ion radiotherapy using cost-reducing cyclotrons and the application of helium is foreseen to increase the interest in this type of radiotherapy. However, further preclinical research is needed to better understand the differential radiobiological mechanisms as opposed to photon radiotherapy, which will help to guide future clinical studies for optimal exploitation of high-LET particle therapy, in particular related to new concepts and innovative approaches. Herein, we summarize the basics and recent progress in high-LET particle radiobiology with a focus on carbon ions and discuss the implications of current knowledge for charged-particle radiotherapy. We emphasize the potential of high-LET particles with respect to immunogenicity and especially their combination with immunotherapy.

## Introduction

Charged particles (CP) are particles with an electric charge, either electrons, protons, or ions. They are produced in linear accelerators, cyclotrons, or synchrotrons for multiple purposes, such as fundamental research and medical applications, i.e., radiotherapy and medical diagnostics. In this review, we will summarize the radiobiological features of heavy ions, i.e., CP with high linear energy transfer (LET), not including protons. These characteristics form the basis of the increasing application of heavy ions in radiotherapy [1], and in new approaches of combined therapies.

A prominent advantage of CP, as opposed to conventional photons, is the inverted dose–depth profile, as they penetrate tissue during radiotherapy. Hence, CP deposit most of their energy in the so-called Bragg peak, i.e., at the end of their trajectory.

The LET is used to describe the deposited energy per track length (unit: keV/µm) and depends on the energy of the individual particle. The LET increases along the path but reaches a maximum only at the end of the particle’s range. Along with this macroscopic difference, the distribution of ionizing events along and inside each particle track is denser on a microscopic scale [2–5]. For irradiation of a given target volume, beams of different energies are superimposed by either passive or active beam shaping, both of which result in a spread-out Bragg peak (SOBP) [4].

The physical properties of protons, worldwide the most commonly used CP in radiotherapy, and heavier ions with higher LET are generally similar. An inverted depth–dose profile is the physical basis of the radiobiological characteristics of CP—protons and heavy ions—on a macroscopic level. However, protons are not considered as high-LET particles. In normal tissue, i.e., the entrance channel of the particle, the LET of protons is close to that of photons and increases only at the very last few microns of the track and outside the SOBP (up to 30 keV/µm beyond the SOBP vs. 2 to 3 keV/µm within the SOBP) [6–8].

Another difference is that heavier ions show a reduced lateral and longitudinal straggling compared to protons. On the other hand, heavier ions feature fragmentation tails beyond the Bragg peak and show nuclear fragmentation in the entrance channel with increasing LET, rendering the peak-to-plateau ratio of the LET unfavorable. For very heavy ions, despite their higher LET, the nuclear fragmentation in the entrance channel leads to an increased dose compared to lighter ions, rendering the peak-to-plateau ratio of the LET unfavorable.

Carbon ions, the second most commonly used particle after protons, rather offer a compromise in that they feature a relatively low LET in the entrance channel (between 11 and 13 keV/µm) and a high LET in the tumor region (between 40 and 80 keV/µm in the SOBP). They are typically applied in energies in a range between 100 and 400 MeV/u in therapeutic conditions and hence require larger accelerator facilities [4, 8–10].

Heavy ions, and in particular carbon ions, bear on top of the inverted dose–depth profile additional biological advantages, i.e., an enhanced relative biological effectiveness (RBE) and a reduced oxygen enhancement ration (OER). On a microscopic level, the basis of these differences is the local density of the ionizing events resulting from the track structure of the ions. The local density of ionizing events increases nonlinearly with LET of the ions until the end of the trajectory, the so-called Bragg peak. Thereby, a larger proportion of complex damage to the DNA is produced, resulting in a higher biological effectiveness for cell killing. Thus, the toxicity of Bragg-peak ions in comparison to low-LET ions in the entrance channel is high. Therefore, a tumor is treated with Bragg-peak ions, while the normal tissue is exposed to low LET ions of the entrance channel. For carbon ions, ideally, the difference in toxicity between the Bragg peak and the entrance channel is high, resulting in best-possible irradiation of the tumor and, compared to conventional radiotherapy, a comparable or even improved sparing of the normal tissue located in the entrance channel [11–13].

Ions other than protons or carbon ions, in particular helium and oxygen ions, are considered for application in charged-particle therapy (CPT) in the future to exploit their special features (see section “Carbon and helium ions in radiotherapy”) [8, 14]. Radiobiological research on effects evoked by high-LET charged particles is of utmost importance to understand the differential effects of low versus high LET (entrance channel versus Bragg peak) in terms of their mechanistic features. This knowledge will help to further improve CPT and develop new innovative approaches. The current knowledge is summarized, and this review will focus on carbon ions, which is currently the most applied form of high-LET particle radiotherapy. However, comparisons to proton therapy will be drawn where appropriate.

## Radiobiology of high-LET charged particles

In addition to advantageous physical characteristics, particularities of the induced biological effects constitute advantages that are increasingly exploited in CPT. Widening of the therapeutic window is a major goal of radiotherapy (RT). A rational to use CP to kill tumor cells is the inverted depth–dose profile and, on top, the enhanced relative biological effectiveness (RBE), lower oxygen sensitivity, and other biological effects.

### RBE in cell studies

**Fig. 1:**
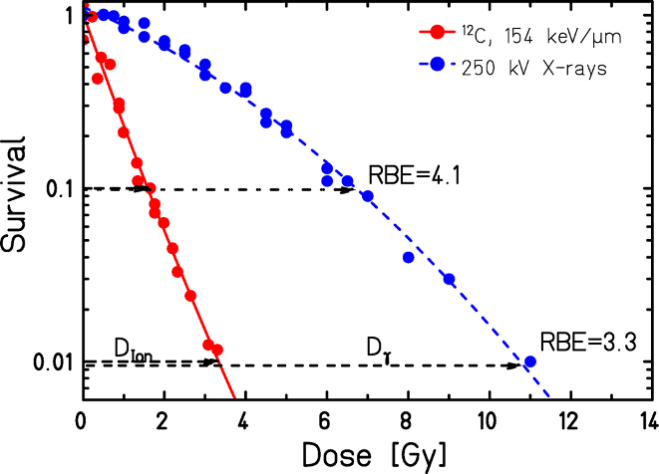
Determining the RBE based on clonogenic cell survival. The graph exemplarily depicts experimental data of clonogenic survival of CHO-K1 Chinese hamster cells. Cells were exposed to photons (250 kV X-rays) or carbon ions (11 MeV/u, 154 keV/µm). The RBE is calculated by considering an isoeffect, e.g., 10% clonogenic cell survival and the subsequent ratio of the doses necessary to obtain the effect (4.1 in this example). As further shown on the graph, the RBE depends on the effect level to be considered, as it varies at different survival levels. The RBE further depends on dose and biological endpoint. (Courtesy of Michael Scholz (GSI Helmholtz Center for Heavy Ion Research GmbH, Germany)); based on experimental data reported by Weyrather et al. [23])

The RBE is the ratio of doses of a reference radiation quality (e.g., photons) to a “test” radiation quality (e.g., CP) needed to achieve a defined level of biological effect, such as clonogenic survival (Fig. 1), cell death, or cytogenetic or other effects that are described in the following paragraphs. The RBE depends on several parameters, such as 1) LET and ion species, 2) biological endpoint, 3) dose or effect level considered, and 4) cell type-specific intrinsic DNA repair capacity and sensitivity. Historically, in vitro studies using cell culture models were dedicated to exploring the RBE of CP systematically, especially for clonogenic survival.

**Fig. 2:**
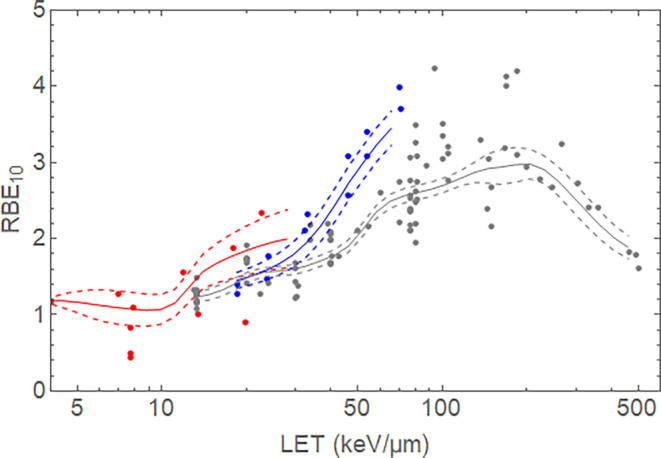
Dependence of the RBE on the LET. The RBE (see Fig. 1) is depicted for different ions and energies with relevance to therapy (*red* protons, data plotted up to 30 keV/µm; *blue* helium ions, data plotted up to 30 keV/µm; *grey* carbon ions, plotted up to 500 keV/µm). The RBE typically rises with an increasing LET up to a maximum (for carbon between 100 and 150 keV/µm) and subsequently drops. Data of clonogenic cell survival (RBE_10_, i.e., at 10% survival) were extracted from the PIDE database (version PIDE 3.3, [15, 24]) with filters for mono-energetic beams and human tumor cell lines. The curve shows a moving average of the RBE values with averaging interval of 0.6 decades using a Blackman window for weighting the considered experimental datapoints, and the error bars depict the standard error of this moving average. (Courtesy of Thomas Friedrich (GSI Helmholtz Center for Heavy Ion Research GmbH, Germany)

1. The RBE increases with LET (Fig. 2) and decreases at higher LET values. The RBE depends at the same LET on the ion species. (Exemplarily shown in [15, 16]). Protons show an increased RBE only at the distal end of the SOBP, but the clinical application is based on an RBE of 1.1 [7, 17–19]. The RBE of carbon ions in the SOBP, where the ions slow down, however, is higher, thus resulting in an increased peak–plateau ratio as compared to protons and a widening of the therapeutic window. Roughly, an RBE ranging between 2 and 3 can be assumed for carbon ions in a therapeutically relevant LET range of 40 to 80 keV/µm (SOBP) [11]. This bears potential especially for the treatment of radioresistant tumors.
2. The RBE depends on the biological endpoint considered, e.g., the RBE for clonogenic survival does not necessarily reflect other endpoints that develop earlier or later in the chronological order of the radiation response [16, 20–22].
3. The RBE values change with dose or effect level considered, as can be inferred for clonogenic survival from Fig. 1. The curves depicting the dose–response relationship of high-LET carbon ions show a larger slope and less curvature, resulting in relatively high RBE values for low doses and in decreasing RBE values with increasing dose.
4. In addition, RBE values for the same endpoint and the same effect level change with the intrinsic radiosensitivity of cells. This means that cells with low radiation sensitivity (corresponding to low α/β ratio) are typically associated with higher RBE values compared to radiosensitive cells. A higher intrinsic radiation sensitivity results in lower RBE values, e.g., due to intrinsic DNA repair deficiencies, as shown in a systematic study in rodent cells [23].

The PIDE database provides a comprehensive collection of RBE data for all ions and cell lines stemming from available in vitro studies [24], using raw data of survival curves and parameterization within the linear quadratic model. The RBE-LET curve for clonogenic cell survival increases up to a certain LET value (for carbon ions between 100 and 150 keV/µm), and then subsequently decreases again due to an overkill effect (for an overview see Fig. 2, [15]). Data resulting from these in vitro studies are the basis of biophysical models used to calculate uniform biological effect, i.e., RBE-weighted doses, in a target volume for therapy.

### Relative biological effectiveness in preclinical in vivo studies

While CP-induced biological effects have been studied widely in vitro, preclinical animal models are more adequate for investigating the normal tissue response and to determine tolerance doses. The peak-to-plateau ratio of RBE-weighted doses in normal tissue and in the tumor for a specific clinical situation reflects the extent of the benefit of CPT for the patient. Although acute and late effects in normal tissue are a dose-limiting factor in treatment, and therefore as or even more relevant than the tumor control, such studies are less frequently carried out.

In skin, spinal cord [25, 26], lung, heart, and vasculature, RBE values for carbon ions of different LET values have been assessed, yielding values ranging from 1.2 (acute skin reaction) to 2.7 (lung fibrosis); the reported RBE values for tumor tissue are comparable (reviewed in [11]). This could be an argument against a beneficial RBE effect of carbon ions, which is expected on top of the clearly advantageous inverted depth–dose profile. However, these are mean values and obtained under very different conditions; the specific ratio of RBE values in normal and tumor tissue depends on the dose per fraction, number per fraction, and the intrinsic radiosensitivities of normal and tumor tissue.

To determine the differential RBE values, animal studies investigating normal and tumor tissue in the same experiment are needed, but these are quite rare. One example is a mouse study investigating the RBE of carbon ions for early skin response and tumor control (fibrosarcoma) [27]. The RBE was found to be higher for tumor control (Bragg peak region, RBE = 2.0–3.0) compared to skin reactions (entrance channel, RBE = 1.2–2.0).

In this study, RBE changes related to dose and number of fractions were investigated. Notably, the decrease of RBE values with dose was different for low and high LET (in line with studies in other tissues such as spinal cord [26]), and less pronounced for tumor compared to skin following high-LET irradiation. This resulted in still advantageous RBE values for normal and tumor tissue after applying high doses per fraction.

The specificity of experimental conditions plays probably a role when comparing studies. In a more recent work, the RBE of carbon ions (SOBP) was assessed in a mouse model with respect to local tumor control (mammary carcinoma), acute skin reaction, and fibrosis, a late effect occurring in skin [28]. In contrast to the first study discussed, the RBE values were quite close for targeted volumes placed in the center or at the distal edge of a SOBP (1.48, 1.36, 1.50), pointing to the absence of a differential RBE as observed in [27].

Radiation effects in tissue and organs depend also on the so-called volume effect, which is determined by the more parallel or serial organization of the functional cells, i.e., by the capacity of tissues or organs to compensate for damaged functional cells and maintain the specific functionality. A typical tissue with a limited volume effect is the spinal cord, where the loss of functional cells can only be compensated when the length of the irradiated segment is small. Mainly in rat models, an extensive RBE database has been created, investigating the occurrence of myelopathy (paresis) for carbon ions (entrance channel, SOBP, LET between 16 and 99 keV/µm), different doses and number of fractions [25, 26, 29]. The results revealed a higher fractionation effect in the entrance channel than in the SOBP, and RBE values increasing with LET [26], notably also for protons at the distal edge of the SOBP [30]. However, a decrease in RBE with dose per fraction can also be inferred from these data, which has also been shown in the acute response of skin and lung fibrosis [11].

To evaluate RBE values for the acute and late responses of skin, mouse models were used, revealing RBE values up to approximately 2 [27, 28, 31]. These values are lower than for myelopathy (spinal cord) and fibrosis (lung), due to the relatively low intrinsic radiosensitivity of skin. However, skin is involved in the exposure of all organs and fibrosis is a major dose-limiting late effect of radiotherapy. At the onset of carbon ion therapy in Germany (GSI), it was important to determine the RBE values for skin, located in the entrance channel of carbon ion therapy. As the response of pig skin resembles much better that of human skin than mouse skin, a study using a minipig model was conducted, revealing similar RBE-weighted dose responses for entrance channel carbon ions and photons [32].

The concept of RBE implies that the same effects are observed after photon and particle irradiation, at different doses for the different radiation qualities. Many of the available animal RBE studies confirm this. However, observations reveal also qualitative differences. One example is a reduced latency time for myelopathy (paresis), which was reported for carbon ion-irradiated spinal cord, in addition to an increased RBE for carbon ions compared to photons [29]. The reduced latency time was discussed to be based on the different quality and reparability of DNA damage induced by carbon ions. However, latency time is perhaps tissue and endpoint specific, as a longer latency time for tumor growth has been reported [33].

### DNA damage patterns and repair

**Fig. 3:**
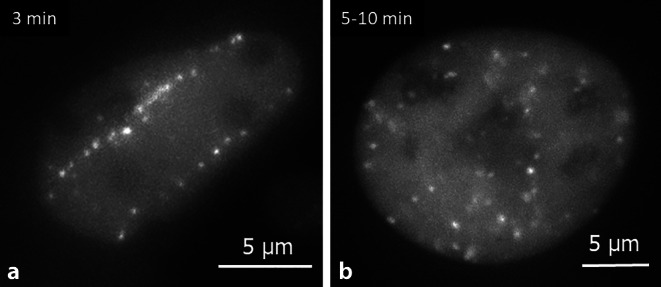
DNA double-strand break repair factor NBS1-GFP is recruited to double-strand breaks induced by HZE ion tracks (**a**) or photons (**b**). Live cell imaging of GFP-tagged NBS1 in U2OS shows the formation of repair foci along the trajectories of the ions (here: 1 GeV/u iron ions, **a**), differing from the pattern of repair foci induced by photons (1 Gy, **b**). (Modified from [55])

For CP exposure, the spatial distribution of ionizing events is different from photons (shown indirectly using DNA damage markers; Fig. 3), and therefore a more frequent occurrence of clustered DNA damage has been predicted by model calculations. This is considered as one main reason for the enhanced RBE of CP for cell death-related effects [34].

Increased occurrence of clustered damage has been shown for particles featuring a higher LET, such as α‑particles [35, 36] or heavier ions [37, 38], but for carbon ions under therapeutic conditions, an increased fraction of clustered double-strand breaks (DSBs) is rather assumed based on models than shown experimentally. In fact, rejoining of DSBs following carbon ions is indeed rather efficient when analyzing the resulting damage via γH2AX [39–41] or premature chromosome condensation [42] assays. Still, in tumors stemming from patients, the 53BP1 repair foci size was found to be increased after carbon ions when compared to photon-treated samples [43]. Recent experimental evidence of clustered DSBs was shown for nitrogen ions, i.e., in conditions not too far from carbon ions; the same study also revealed a dependence on the cell type [44]. Hence, despite featuring a broader distribution than for α‑particles, considering also biophysical modelling [45] and in vitro data [46–48], an increased fraction of clustered DNA damage as compared to photons or protons can be assumed for carbon ions (reviewed in [49]).

As a consequence, the DNA damage response following CP exposure is different for that of photons and constitutes a mechanism underlying the higher RBE of CP. The two main pathways to repair DSBs are homologous recombination (HR), active only in the S and G2 phases of the cell cycle, and non-homologous end joining (NHEJ). Resection is a fundamental part of HR but does not occur in canonical NHEJ in G1. Upon induction of complex DNA damage, it is hypothesized that the damaged cells engage error-prone, alternative DNA repair pathways (reviewed in [50]). This is endorsed by the reported increase of resection with LET in G1 phase cells [51]. Thus, these error-prone repair pathways become prevalent in contrast to the canonical non-homologous end-joining pathway, which is predominantly used after low-LET irradiation [39, 50–53].

Also different upon CP exposure compared to photons are the kinetics of repair and regulatory protein recruitment [54]. Differences in the recruitment of regulatory proteins were observed comparing simple and complex DSBs, as well as sites of single versus clustered DSBs [55]. A relocation of damage from the initial induction site to the periphery of the heterochromatin and a fast recruitment of repair and regulatory proteins to heterochromatic lesions inside murine chromocenters have been reported [56]. The differences in DNA damage and subsequent repair lead to higher frequencies of false repair of CP-induced lesions, which is reflected in a higher yield of mutations and cytogenetic damage.

### Cytogenetic damage

Chromosomal aberrations are observed both in vivo and in vitro after carbon ion exposure [57]. The RBE-LET curve peaks around 100–200 keV/µm, with a subsequent decrease [58, 59]. The higher complexity of the DNA lesions renders them less repairable or results in false repair, thus leading to a higher complexity of chromosomal aberrations after CP exposure [37, 59–62]. However, a clear difference to photons is only observed for high-LET radiation qualities such as low energy alpha particles and iron ions, i.e., with a higher LET than therapeutic SOBP carbon ions as delivered in the tumor region [63, 64]. It is important to point out that classical methods of cytogenetic analysis require the transition through mitosis, using metaphase chromosomes or micronuclei [65]. This can lead to an underestimation of chromosomal damage with higher LET exposure. A partial technical solution to this is the technique of premature chromosome condensation [66–68]. The underestimation is mainly due to the arrest of proliferating cells that prevents a transition through mitosis. This effect increases with LET, as described in section “Cell death and other cellular responses” [69, 70].

This plays a minor role when assessing chromosomal aberrations in normal tissue following low LET exposure, corresponding to the entrance channel during treatment, because the analysis is, if at all, only marginally affected by an arrest in cell cycle progression. In circulating blood cells of treated patients, lower or at least similar frequencies of chromosomal aberrations were detected for carbon ion compared to patients treated by photons (IMRT) [71–73]. An additional aspect is that the volume effect turned out to be more important, showing a pronounced difference when comparing small- versus large-field IMRT treatment [73]. Furthermore, the fraction of damaged lymphocytes was lower after carbon ion compared to X‑ray treatment, and the radiation-induced decrease in the number of white blood cells and lymphocytes, a common sequela of radiotherapy, was less pronounced after carbon ion therapy. In line are in vitro results obtained in hematopoietic stem and progenitor cells following exposure to carbon ions and photons [74]. Taken together, these results suggest that CP, i.e., low-LET carbon ions occurring in the entrance channel, induce a comparable level of cytogenetic damage compared to photons in the normal tissue, while the yield of complex damage, mutations, and cytogenetic rearrangements is expected to be higher in the Bragg peak [75]. This is endorsed in studies investigating the level of micronuclei, arising from the loss of parts of or whole chromosomes, which is an established marker of cancer risk [76]. In tumor cells, the regulation of cell cycle progression is reduced or abrogated, and a more effective induction of micronuclei following high-LET CP exposure, compared to similar doses of low-LET CP or photons, was observed [77–82]. In a mouse model of squamous cell carcinoma, RBE values for micronuclei were determined to increase to a maximum at an LET of 192 keV/µm, ranging between roughly 8 and 4 for different oxygen conditions [79]. Cytogenetic damage and mutagenicity, in turn, constitutes part of the immunogenicity and may foster a favorable immune response in vivo (see section “High LET particles in the context of immunogenicity and combined therapy”) [75].

### Cell death and other cellular responses

The cellular fate after irradiation is not exclusively influenced by induction and repair of DNA and chromosomal damage, but also via molecular responses that are either cytoprotective, cytostatic, or cytotoxic [83]. Regarding cytotoxic cellular responses, apoptosis and necrosis are the major cell death pathways described after irradiation, preceded under specific circumstances by mitotic catastrophe. Autophagy is a cytoprotective process related to restoring and preserving cellular viability. Carbon ions were shown to be able to induce autophagy even more efficiently than photons [84, 85]. A cytostatic response is the inhibition of cell cycle progression in proliferating cells. More details related to these processes as part of the general response to CP exposure will follow.

The maintenance of genomic integrity by different inactivation mechanisms is a feature of normal cells, although also occurring in tumor cells in a reduced number of cells or in an incomplete way. In vitro experiments comparing carbon ions or α‑particles with photons in many cell types of normal tissue and in tumor cells revealed that all modes of inactivation are more pronounced with increasing LET, as follows [16, 69, 70, 86–92].

The cell cycle arrest is considered to allow for repair of radiation-induced damage and to prevent the transmission of genomic alterations to daughter cells. An alternative process to cell cycle arrest to maintain genomic integrity is cell death. Which molecular pathway is engaged directly after radiation insult is cell type specific and depends on the reparability of the damage. Radioresistent cells often carry mutations in the p53 gene, coding for a key protein of the cellular arrest in G1 phase. Instead, a delay or arrest in G2/M phase of the cell cycle is induced, which is more pronounced after exposure to carbon ions compared to photons [86, 87]. It is again cell type specific whether the release from the cell cycle block leads to cytotoxic (cell death) or further cytostatic mechanisms, non-lethal processes like senescence (terminal cell cycle arrest), premature differentiation of proliferating progenitor into functional cells, or mitotic catastrophe.

One cytostatic mechanism in response to irradiation is premature differentiation, which is set on in many normal cell types and has been shown, for example, in fibroblasts [16] and keratinocytes [92]. In human fibroblasts, RBE values were determined in vitro for premature differentiation and other parameters. Enhanced premature differentiation is a survival strategy after radiation damage and its yield is dose and LET dependent. In contrast to premature differentiation, the onset of senescence, which is terminal cell cycle arrest, occurs in normal, in particular mesenchymal and epithelial cells, as well as tumor cells, i.e., glioblastoma and head and neck squamous cell carcinoma (HNSCC), to a similar extent after low-LET carbon ion exposure compared to after photons [86, 92, 93]. It is more pronounced after proton [94] or high-LET irradiation as compared to photons [91, 93, 95]. In tissue, senescent cells can induce inflammation by the release of cytokines. This inflammatory phenotype, i.e., senescence-associated secretory phenotype (SASP), is considered as cancer promoting in association with tumor cells. In contrast to the higher efficiency of carbon ions in inducing senescence, the induction of SASP in HNSCC was reported to be at a similar level as for photons [95, 96].

With respect to a global inflammatory response, the observations are not consistent and may be cell type specific. Low-LET carbon ions induce a similar response compared to photons in skin cells and tissue equivalents [92], but a more pronounced response in leukocytes (peripheral blood mononuclear cells) [97] compared to photons. Surprisingly, for even lower LET, i.e., proton exposure, a qualitatively and quantitatively different response compared to photons has been reported [98]. Overall, the LET dependence of effects in the tissue that play a role in inflammation is not fully clear. A deeper understanding is needed, as inflammation plays an important role in adverse effects in normal tissue.

As already mentioned above, upon induction of irreparable damage, an alternative to the cytostatic mechanism is the onset of cytotoxic pathways leading to cell death. A higher efficiency following high-LET exposure has been demonstrated in terms of percentage of cells undergoing apoptosis [99]. Mitotic catastrophe is considered another cytostatic mechanism in case of impaired mitosis and often precedes the occurrence of cell death. Markers for mitotic catastrophe are giant nuclei and multinucleation, as well as the formation of micronuclei [100]. Only one study is available on carbon ions, reporting it to be enhanced compared to photons [101].

Relatively little has been investigated in this context, but the different patterns of cell inactivation reveal the potential for investigations on combination therapies of, e.g., immunotherapies or targeted therapies with CPT, different from conventional radiotherapy. Important in this respect is—as for cell cycle arrest—the status of p53, a major inducer not only of cell cycle arrest but also of apoptosis. Following exposure to carbon ions or heavier ions, apoptosis is less dependent on the p53 status compared to photons [102–104]. In addition, p53-independent apoptotic pathways are involved to a higher greater after high-LET irradiation [105, 106], for example the ceramide pathway [107]. For further details we refer to a comprehensive review by [108].

Based on the observation that higher photon doses induce also necrosis [109, 110], it can be assumed that high-LET irradiation promotes necrosis. This has been demonstrated for regulated necrosis, i.e., necroptosis and ferroptosis [111, 112]. However, a quantitative comparison with photons, which induce these types of cell death [113, 114], is pending. These are first hints that CP with higher LET do not only induce more apoptosis than photons, but also set on more intensively other cell death pathways such as regulated necrosis.

### Hypoxia

Hypoxia, whether chronic or acute, is associated with poor clinical prognosis and about 50 to 60% of all solid tumors feature hypoxic regions, rendering the tumors a more aggressive phenotype and significantly affecting the outcome of RT [115]. The impact of low oxygen concentrations on the efficiency of RT is based on the reduced formation of reactive oxygen species (indirect radiation effect), thus conferring radioresistance. The oxygen enhancement ratio (OER), i.e., the ratio of doses necessary to inactivate tumor cells in hypoxic vs. oxic conditions, can be as high as 3, rendering tumor control difficult [116, 117]. RT often enables reoxygenation of the remaining tumor tissue due to the classical fractionation regimen. However, hypoxia still negatively affects the outcome of RT, being a problem especially in hypofractionated regimens, where reoxygenation is intrinsically limited [118–121]. Since for heavy ions, as opposed to photons, the direct radiation effect is dominant, the effects depend less on ROS production and hence oxygen concentration. Therefore, heavy ions are considered as a tool to overcome hypoxia, optimally reducing the OER to a value of 1.

Indeed, the OER depends only minimally on the dose, but rather more on the LET and on the oxygen concentration in tissues (partial oxygen pressure, pO_2_) [122, 123]. The dependence on oxygen concentration and LET has been intensively studied, mostly in vitro, while in vivo data are scarce. A detailed in vitro study showed that the LET dependence of the OER has a similar trend for different hypoxic conditions but decreases to the value “1” only at LET values higher than 200 keV/µm [124]. However, for carbon ions under therapeutic conditions, this is only reached at the distal end of the SOBP [8]. In vitro measurements also show that acute hypoxia induces radioresistance to a higher extent than chronic hypoxia. Acute hypoxia has been suggested to result in more aggressive tumor phenotypes [115]. Interestingly, for carbon ions, no significant differences in the radioresistance have been shown between acute and chronic hypoxia [125, 126], indicating a further advantage for carbon ion therapy in the treatment of hypoxic tumors.

Hypoxia can be studied in vivo by using animal models with clamped tumors, thus interrupting blood and oxygen supply [127, 128]. Older studies induced hypoxia by giving the animals nitrogen gas to breathe shortly before tumor exposure in vivo, then sacrificing the animals and subsequently measuring cell survival ex vivo (e.g., [129]). These differences in methodology, however, demonstrate the limitations of comparability between the few in vivo studies available.

Reduced OER values were reported for carbon (1.9) and neon beams (1.7) as compared to photons (2.2), and the authors associate the reduced values with the increasing LET (70–120 and 115–240 keV/µm for carbon and neon, respectively) [129]. A study reported hypoxia induced by clamping in a rat prostate cancer model resulting in an increase of 15% in the dose necessary to control the tumor (TCD_50_) for photons, but no differences in the TCD_50_ following exposure to carbon and oxygen ions (dose-averaged LET 65 and 101 for carbon and oxygen, respectively) [130]. In a study comparing carbon ions and photons using a clamped tumor mouse model, a small decrease in the OER along the SOBP was reported for carbon ions [127]. Of note, reoxygenation in tumors of mouse and rat models appeared faster after exposure to carbon ions than after exposure to photons [128, 131, 132], and in one study this was related to increased microvascular density [128], thus providing further rationale for treatment of hypoxic tumors with carbon ions. Of note, defined conditions of the oxygenation levels in in vitro experiments render the comparison to in vivo studies, where the oxygenation level cannot be strictly controlled, difficult. Additionally, the complexity of the in vivo models is enriched by the tumor microenvironment and immune responses, and clamping may induce effects beyond limitation of oxygenation [130].

Hypoxic conditions induce hypoxia-inducible factors (HIFs), transcription factors promoting tumor progression through various mechanisms [133], and these are pharmacological targets in cancer therapy [134, 135]. Interestingly, both in vitro and in vivo, carbon ions were reported to attenuate HIF expression [136–139].

As for clinical evidence of the effectiveness of carbon ions in hypoxic tumor treatment, only one study individually measuring pO_2_ in uterine cancer patients has so far demonstrated reduced radioresistance and, hence, enhanced effectiveness of carbon ions [140]. However, in locally advanced pancreatic cancer, a tumor reported to be highly hypoxic [141], carbon ions showed promising clinical results (for an overview see [142]), which could be attributed to a reduced OER.

## High-LET particles in the context of immunogenicity and combined therapy

The ultimate aim of cancer radiotherapy is induction of cell death to kill or inactivate tumor cells, including the fraction of stem cells in the target volume. However, in the past two decades, it has become increasingly evident that the success of RT depends largely on the immune status and the immune responses elicited by irradiation. Combined treatments have been proposed, investigated in preclinical studies, and are now being tested in clinical trials. Irradiation acts by rendering the tumor accessible to recognition by the immune system, i.e., enhancing the immunogenicity of the tumor and fostering the subsequent immune response.

As discussed above, CP display the advantage of an enhanced RBE in killing tumor cells, and most likely modify the pattern of cell death compared to photons. Both influence the immunogenicity of the tumor, which, in turn, is specific to the immune system of the host and depends on the tumor microenvironment [83]. The immunogenicity of the different mechanisms of cell death is determined by the

**Fig. 4:**
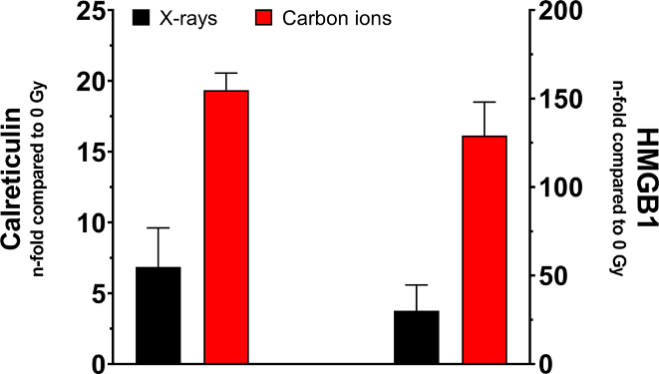
Adjuvanticity of carbon ions assessed in osteosarcoma cells in vitro. The graph depicts exemplarily the translocation of calreticulin and release of HMGB1 (measured by flow cytometry and ELISA, respectively) 48 h following exposure to X‑rays or carbon ions (SOBP, 75 keV/µm). At the same physical dose (8 Gy), carbon ions show an increased effectiveness in inducing DAMPs compared to X‑rays (own unpublished results)

1. antigenicity, i.e., the radiation-induced neoantigen repertoire enhancing the mutational burden of the tumors with low antigen burden and functioning as targets for CD8+ cells [143]. Linked to the induction of clustered DNA damage, CP are more effective than photons in the induction of mutations [144], induce a different quality of mutations [145], and chromosome aberrations [59], bearing the potential for more efficient support of an anti-tumor immune response. Related to the different induction of DNA damage is the occurrence of small DNA fragments in the cytoplasm with subsequent activation of stimulator of interferon genes (STING) and subsequent induction of type I interferon (IFN) and stimulation of the maturation of dendritic cells [146–148]. The influence of CP exposure remains to be elucidated [149].
2. adjuvanticity is the spatiotemporal release of danger signals (damage-associated molecular patterns, DAMPs). Danger signals, i.e., ATP, calreticulin (CRT), and high-mobility group box 1 (HMGB1) constitute components of immunogenicity, leading to the recruitment and maturation of antigen-presenting cells (APCs) [83, 150]. Only a few studies are available on the impact of CP, with conflicting results [151–155]. One study reports an increase of HMBG1 release with LET [156]. This result is confirmed in our own unpublished data, with enhancement upon carbon ion exposure compared to X‑rays for both HMGB1 release and CRT surface translocation (Fig. 4).

CP might differentially affect the cellular responses and, in particular, the induced cell death pattern following exposure that leads to a modification of immunogenicity. Several forms of cell death, i.e., apoptosis, ceramide-mediated death, necrosis, necroptosis, or ferroptosis, were shown to be triggered (in part more efficiently) by carbon ions [85, 86, 102, 107, 111, 112]. The choice between cell death and other forms of cell inactivation, i.e., autophagy, cell cycle arrest, cell differentiation, and senescence, could also play a role, especially when they are affected differently by CP exposure compared to photons. In addition, differences in the tumor microenvironment contribute, and the sparing of circulating immune cells, in particular lymphocytes, by CP compared to photon exposure (for further details, we refer to [157]).

The results reported so far underpin the potential of CPT, especially of carbon ion radiotherapy (CIRT) with respect to adjuvanticity and a putative subsequently enhanced immune response. Preclinical studies on the impact of a combination of immunotherapy with CPT are scarce but promising, although the results turned out to be highly dependent on the preclinical models used. An injection of pretreated dendritic cells (DCs) stimulated the activity of CD8+ T cells in combination with carbon ions [158, 159], but no comparison to photons was performed. Besides, in one study directly comparing to photons, improved control of the primary and the abscopal tumor and reduction of lung metastasis was observed in an osteosarcoma model after exposure to isodoses of SOBP carbon ions in combination with either checkpoint inhibitors [160, 161] or injection of pretreated DC [152]. These results underline the potential of metastatic suppression reported for carbon ions [162]. Furthermore, CP exposure leads to a reshaping and more pronounced infiltration of immune cells. For example, a higher number of CD8+ T cells infiltrating into the tumor and improved survival were observed in melanoma models [154], and higher frequencies of activated naïve T cells were observed infiltrating an abscopal tumor [163].

## Discussion

This review is dedicated to the radiobiology of high-LET charged particles and their use in radiotherapy. The physical characteristics of charged particles determine the specific biological effects, in particular the inverted dose–depth profile, resulting in an improved volume conformity of charged particle irradiation compared to photons. This accounts also for protons. Moreover, for heavier ions an enhanced RBE can be exploited in the tumor region, whereas in the entrance channel a lower RBE brings along the potential for sparing dose in the normal tissue and a resulting higher safety.

### Relative biological efficiency

The basis for a clinical application of higher LET CP was established by radiation biology. An important tool was created by the definition of the RBE, which allows relating the biological effects of CP with photons. In particular, to obtain differential RBE values for effects in tumor and normal tissue, systematic studies were first performed in vitro in tumor and normal tissue cells. These studies provided important insights, especially for clonogenic survival, but also for DNA repair, cytogenetic effects, cell death, and other processes. These effects bear not only quantitative but also qualitative differences for CP compared to photons, and, as a consequence, constitute a mechanism underlying the higher RBE of CP. One example is the DNA damage response, which is different due to the more frequent occurrence of clustered damage and less reparability of the damage following CP exposure.

As can be inferred from the PIDE database, the RBE for the same LET is higher at low doses than at higher doses (Fig. 2, [15]). This would be disadvantageous for the radiation treatment of tumors, because lower doses occur in normal tissue, while higher doses occur in the tumor. However, to estimate the differential RBE in normal and tumor tissue, a consideration of several factors is necessary. In a radiation treatment, normal tissue is located in the entrance channel and irradiated with low-LET carbon ions, whereas tumor tissue is located in the Bragg peak region and irradiated with carbon ions of higher LET. Indeed, the RBE depends on radiation LET, dose, and dose per fraction. Furthermore, the specific radiation sensitivities (i.e., α/β ratios) of the tissues plays a role. These aspects are better addressed in preclinical in vivo studies, allowing for a better consideration of tumor control and normal tissue toxicity.

### Carbon and helium ions in radiotherapy

As described above, the currently used particles in radiotherapy, mainly protons and to a lesser extent carbon ions, represent compromises and each have their advantages and drawbacks. With respect to new perspectives to circumvent inconvenient fragmentation, helium is a prominent candidate ion in cancer treatment different from protons or carbon, since it features tradeoffs between the two. For instance, as compared to protons, helium ions result in reduced lateral and longitudinal straggling and have a slightly enhanced LET (ranging from about 4 to 40 keV/µm), which results in an increased RBE (experimentally, roughly ranging between 1.3 and 3) [14]. The physical advantages in comparison to carbon ions render helium ions particularly interesting for treatment of pediatric cancer disease [164]. Early studies revealed excellent results with respect to local tumor control [165], and the first worldwide patient so far was treated with an active scanned helium beam at HIT in compassionate use [14].

### Clinical studies

An increasing amount of long-term follow-up data from studies using CIRT show efficacy and safety, i.e. reduced toxicity of the treatment [9]. The two major indications for treatment of cancer with CIRT are radioresistance and close vicinity to sensitive organs at risk. The list of cancer entities treated with CIRT is nowadays long (for a comprehensive review, we refer to Malouff and colleagues [1]). Some of the tumor entities where CIRT has been successfully applied comprise high-grade gliomas, skull base chordoma and chondrosarcoma, osteosarcoma, adenoid cystic carcinoma, non-small cell lung cancer, and pancreatic cancer.

Sparing of healthy tissue and reduced toxicity is especially important in patients with a long life expectancy, in particular for pediatric patients. Therefore, particle therapy with carbon ions, which is known to have a higher RBE also for late effects such as carcinogenesis [166], is classically considered too risky. Nonetheless, data from pediatric patients treated at centers in Japan (NIRS) and Germany (GSI, HIT) with CIRT (mainly skull base tumors, head and neck tumors, or osteosarcomas) showed promising local control and favorable results with respect to toxicity and, interestingly, no significant differences in toxicity upon comparing protons and carbon ions were reported [167–170]. Consistently, CIRT has been proposed as a promising tool for the treatment of pediatric cancers [171].

Along this line, a retrospective analysis of a cohort of 1580 prostate cancer patients treated with CIRT at NIRS revealed a lower risk of second cancers than in patients treated with photons [166, 170]. This is endorsed by dosimetric measurements, demonstrating that doses from secondarily formed neutrons are lower using CIRT with active scanning compared to Intensity modulated radiotherapy (IMRT) or passive scattering beams [172–174].

When it comes to clinical data on CIRT, however, two major problems arise. One is the lack of data stemming from randomized clinical trials comparing CIRT to photons or protons. A few randomized studies are currently ongoing, with the perspective to allow for better comparison (for an overview, we refer to [11]). Moreover, the studies often lack data on whether improved results for CIRT are due to higher volume conformity and hence better tissue sparing, or whether the higher RBE may evoke different biological mechanisms. The latter is of particular interest when it comes to the immunogenic effects of radiotherapy (see above). Mechanistic investigations integrated in a clinical study are of interest. For example, one study with biomedical investigations is ongoing (ICONIC, NCT05229614, [175]).

### Perspectives and innovative approaches

Despite promising results for carbon ion therapy, there is room for improvement, e.g., with respect to overcoming cancer-specific radioresistance, tackling metastatic cancer disease, and further reducing normal tissue toxicity. The radioresistance of tumors is typically associated with hypoxia, but has also been attributed to immunosuppressive tumor microenvironment (TME) as, for example, in pancreatic ductal adenocarcinoma [176, 177]. With respect to the immunosuppressive TME, as discussed above, CIRT can be beneficial due to an increased immunogenicity. The latter remains to be elucidated; however, preclinical research points to biological advantages. For example, a reduced metastatic load has been reported following exposure to CIRT [152, 161], but it is unclear whether the effects stem from better inactivation of tumor cells and subsequently less metastatic dissemination or from a systemic immune response triggered by CIRT. Moreover, the physics of CIRT, i.e., the sparing of healthy tissue and hence cycling immune cells, as well as a putatively reduced lymphopenia [178], render it a good match with any combination of immunotherapy.

The fractionation schemes and the timing of application of drugs and irradiation remain a challenge of the combination of immunotherapy with CP [179]. Moreover, new innovative protocols suggest the extension of time between fractions (ultrafractionated stereotactic adaptive radiotherapy, PULSAR [179]), which could allow for improved synergy with immune therapy and might be an interesting approach using CP.

Preclinical studies have shown that the OER is reduced with increasing LET and reoxygenation occurs faster—going along with increased microvascular density—and the expression of HIF, a molecular factor conferring radiation resistance under hypoxic conditions, is attenuated. Taken together, this provides a good rationale for treatment with CIRT and an interest in comparative clinical studies [142, 180, 181]. Hypofractionated CIRT plus concurrent gemcitabine [10] at NIRS showed remarkable results obtained for locally advanced pancreatic ductal adenocarcinoma, a highly hypoxic tumor, which were confirmed in further trials [182, 183]. However, further perspectives to better overcome the radioresistance conferred by hypoxia are new ions beyond carbon ions, i.e., oxygen ions, with a higher RBE and lower OER [8]. The higher RBE brings along the disadvantage of increased toxicity in normal tissue [184], excluding the application of neon or argon ions, which showed higher toxicity and severe side effects in early patient studies in Berkeley [185]. However, with recent advances in minibeam irradiation (spatial fractionation, see section “New technical approaches”), the problem of increased toxicity can be addressed, as shown in a preclinical study [186]. To overcome hypoxia, an increased LET in the tumor region is favorable to reduce the OER as close as possible to one. As suggested in some studies [187, 188], a compromise can be a boost treatment of the tumor regions identified as hypoxic [189–192] with oxygen ions, taking advantage of an increased RBE and reducing the toxicity at the same time, since only a boost of high-LET ions is delivered. A combination of various ion species within the same treatment is considered in this context [193–196]. Also new protocols for selective targeting of hypoxic, immunosuppressive tumor segments, i.e., stereotactic body radiation therapy based partial tumor irradiation targeting hypoxic segment of bulky tumors (SBRT-PATHY) would perfectly fit with CPT [197].

### New technical approaches to reduce normal tissue toxicity

In-depth knowledge of the biological mechanisms of increased RBE is critical for reducing normal tissue toxicity. Because of preclinical research and clinical studies, protocols and applications of CPT have improved [166]. Irrespective of the radiation quality, also efforts to find technical solutions have been continuously undertaken and techniques such as IMRT, arc therapy, and others are now widely used in clinics. One new radiotherapy approach for reducing normal tissue toxicity is FLASH therapy, mostly assessed with electron beams. The term “FLASH” originates from the English “lightning.” In the context of radiotherapy, it means the delivery of (high) doses with an ultra-high dose rate (minimum 40 Gy/s). First observations go back to the 1960s [198], and preclinical research in the past decade has confirmed that FLASH-RT is less toxic for normal tissues compared to conventional RT, while as effective as conventional RT for tumor control.

The high dose rates of FLASH are discussed as an alternative to the respective conventional dose rates also for heavier ions such as carbon ions, with the potential to even further enlarge the therapeutic window compared to electron and even proton FLASH [199]. However, the exploration has only begun. In one study using carbon ions with FLASH dose rates, not only the control of the primary and an abscopal tumor was improved, but also the reduction of distal metastasis in unexposed lungs [200, 201].

Challenges to the clinical application of carbon ion FLASH are so far technical issues, i.e., for beam delivery and dosimetry. Laser-driven accelerators deliver ultrashort pulse particle beams, discussed to be an ideal tool for the investigation of biological effects of high dose rates and their application in radiotherapy [202]. Current solutions and their experimental validation are discussed in [203]. A remaining drawback is, irrespective of the radiation quality used, that the underlying radiochemical and biological mechanisms are not yet clear.

The potential of FLASH dose rates combined with CP, in particular heavy ions, for tissue sparing needs more mechanistic elucidation to be fully exploited. A comprehensive review of the current knowledge has recently been published [199].

One emerging approach is irradiation by spatial fractionation using micro- or minibeams of X‑rays or protons to obtain improved sparing of the normal tissue. With this technique, passively beam-shaping devices, i.e., blocks or multileaf collimators, deliver a grid-like pattern of irradiation with an inhomogeneous distribution of dose in the irradiated tissue. This distribution of dose is described by the ratio of the “valley dose” (cold spots) and the “peak dose” (hot spots).

The use of a proton minibeams revealed an efficient sparing of normal tissue, for example in a glioblastoma rat model [204] or in a mouse model [205, 206]. Studies using heavier ions are scarce. However, for lithium ions, comparable toxicities in terms of cognitive impairment have been reported for minibeam and conventional irradiation of rat brains that have been positioned in the entrance channel of the beam [207]. As for FLASH, also for minibeam irradiation are the underlying mechanisms not yet clear.

## Conclusion

Due to well-understood physical differences of CP as compared to photons, the biological efficiency in cell killing is enhanced and this is exploited in current CPT. However, differential effects have been reported beyond cell killing. For instance, DNA repair pathway choice, the induced damage pattern, the remaining cytogenetic damage, or the cell death pathway choice may well differ for high-LET particles. These bear potential for synergies especially for combination therapies, most prominently with immunotherapy, as an increased immunogenicity for high-LET particles has been anticipated and reported in preclinical studies. There is continuous development in the field of CPT and the use of high-LET ion species apart from carbon ions (mainly oxygen and helium ions) has capacity to move forward in yet unresolved problems of radiotherapy, for example hypoxia or CPT treatment for pediatric cancers. Furthermore, new approaches and concepts of dose delivery with respect to spatial distribution and dose rate are under investigation to enlarge the therapeutic window by reducing normal tissue toxicity. With respect to the aim of enlarging the therapeutic window, which especially includes the fields of hypoxia, dose delivery at high dose rates (FLASH), normal tissue effects, and immunogenicity for combination therapies require an increased research effort. The increasing number of centers for CPT around the world and new technology reducing the high costs of CPT are indicative of the growing interest in the field. The radiobiology of high-LET particles definitely deserves more investigation to better exploit its potential for cancer treatment.
